# Polymer models of yeast *S. cerevisiae* genome organization

**DOI:** 10.1186/1756-8935-6-S1-P128

**Published:** 2013-04-08

**Authors:** Geoff Fudenberg, Jon-Matthew Belton, Anton Goloborodko, Maxim Imakaev, Job Dekker, Leonid Mirny

**Affiliations:** 1 Biophysics, Harvard University, Boston, MA, 02115, USA; 2 Systems Biology, UMASS Medical School, Worcester, MA, 01605, USA; 3 Physics, MIT, Cambridge, MA, 02139, USA; 4 Division of Health Science and Technology, Harvard-MIT, Cambridge, MA, 02139, USA

## Introduction

Three-dimensional (3D) chromosomal organization impacts critical cellular processes including transcription, replication, and genomic stability. Despite the ubiquity of these challenges, recent 3C-based experiments [1] suggest that major features of interphase chromosomal organization vary across eukaryotes. In human cells, Hi-C revealed that chromosomal regions of similar functional state (eg. high gene expression) were enriched for contact probability in 3D [2]. Conversely, 3D contact probability was depleted between regions with different functional states. Moreover, the probability of a contact between two genomic loci was found to be inversely proportional to intervening genomic distance, *s*^1^ and consistent with an unknotted non-equilibrium fractal globular polymer state. Here we analyze new, high-coverage, Hi-C data and developed stochastic simulations of polymer dynamics to study the spatial organization of yeast chromosomes.

## Results

Unlike in human chromosomes, we find no evidence of a domain-type organization in yeast. Furthermore, we find that contact probability decays more like *s ^-^*^3/2^ with increasing genomic distance in *cerevisiae;* this indicates that yeast chromosomes do not exhibit a fractal globule organization. Instead, we find that a Rabl-like organization of chromosomes and constraints from the nucleolus appear to be the most prominent features of chromatin organization [3,4]. In this Rabl organization, centromeres are co-localized near the spindle pole body on one side of the nucleus. Our stochastic polymer simulations allow us to reconstruct conformational ensembles consistent with Hi-C maps. We demonstrate that a Rabl organization leads to cross-like patterns of interactions between centromeric regions, as observed in yeast Hi-C data. Our simulations also allow us to match experimental data on diffusion of individual genomic loci; this allows us to study the temporal evolution of chromosomal conformations.

## Conclusions

Our models show that: (1) yeast chromosomes are generally consistent with a mildly confined “equilibrium globular” polymer state, in contrast with observations in human cell lines; (2) tethering in a Rabl conformation induces a ‘polymer brush’ effect which reproduces the majority of observed intra- and inter- chromosomal Hi-C interactions in yeast; (3) rapid progression through the cell cycle allows for spatial, but not necessarily topological, equilibration of yeast chromosomes, limiting their mutual entanglement.
